# Conjugation Inhibitors and Their Potential Use to Prevent Dissemination of Antibiotic Resistance Genes in Bacteria

**DOI:** 10.3389/fmicb.2017.02329

**Published:** 2017-11-30

**Authors:** Elena Cabezón, Fernando de la Cruz, Ignacio Arechaga

**Affiliations:** Instituto de Biomedicina y Biotecnología de Cantabria (IBBTEC), CSIC-Universidad de Cantabria and Departamento de Biología Molecular, Universidad de Cantabria, Santander, Spain

**Keywords:** antibiotic resistance, type IV secretion systems, inhibitors, fatty acids, bacterial conjugation

## Abstract

Antibiotic resistance has become one of the most challenging problems in health care. Bacteria conjugation is one of the main mechanisms whereby bacteria become resistant to antibiotics. Therefore, the search for specific conjugation inhibitors (COINs) is of interest in the fight against the spread of antibiotic resistances in a variety of laboratory and natural environments. Several compounds, discovered as COINs, are promising candidates in the fight against plasmid dissemination. In this review, we survey the effectiveness and toxicity of the most relevant compounds. Particular emphasis has been placed on unsaturated fatty acid derivatives, as they have been shown to be efficient in preventing plasmid invasiveness in bacterial populations. Biochemical and structural studies have provided insights concerning their potential molecular targets and inhibitory mechanisms. These findings open a new avenue in the search of new and more effective synthetic inhibitors. In this pursuit, the use of structure-based drug design methods will be of great importance for the screening of ligands and binding sites of putative targets.

## Introduction

Antibiotic resistance is becoming a major threat for human health (WHO, 2014). Widespread abuse of antibiotics in human health and food production are threating our current health systems and challenge future care (Giske et al., 2008; Boucher et al., 2009; Rice, 2009; Chang et al., 2015). However, despite these threats, few new antibiotics are becoming available to fight against multi-resistant bugs (Gould and Bal, 2013; Hede, 2014). Bacterial conjugation is one of the main mechanisms whereby bacteria become resistant to antibiotics (Mazel and Davies, 1999; Waters, 1999). Thus, the search for specific conjugation inhibitors (COINs) is a foremost concern in the fight against the spread of antibiotic resistance genes (Smith and Romesberg, 2007; Baquero et al., 2011; Baym et al., 2016). In this pursuit, several compounds were reported to inhibit bacterial conjugation specifically, although most turned out to be unspecific growth inhibitors (Michel-Briand and Laporte, 1985; Hooper et al., 1989; Conter et al., 2002; Lujan et al., 2007; Nash et al., 2012).

Bacterial conjugation is a mechanism by which DNA is transferred between two bacterial cells. The process consists of two steps. In a first stage, DNA is mobilized by a set of proteins, encoded by MOB genes. In a second step, DNA is transported across a secretion system [type IV secretion system (T4SS)] (de la Cruz et al., 2010; Cabezón et al., 2015). T4SS is a complex formed by proteins encoded by another set of genes, also known as MPF (mating pore formation) genes (Fernandez-Lopez et al., 2006). Conjugative Gram negative bacteria usually contain three MOB genes encoding proteins involved in DNA processing (Smillie et al., 2010). The most ubiquitous of these genes encodes a relaxase protein, which cleaves one of the plasmid strands at the origin of transfer (oriT) (Garcillán-Barcia et al., 2009) (Supplementary Figure S1A). Upon this, the relaxase protein remains covalently bound to the DNA at the 5′ end. This nucleoprotein complex is recruited at the secretion channel (T4SS) with the assistance of the coupling protein, an ATPase present in most conjugative plasmids (Llosa et al., 2002; Tato et al., 2005) (Supplementary Figure S1B). A third, accessory protein is usually involved to ensure the correct DNA folding for the relaxase action (Moncalian and de la Cruz, 2004).

Conjugative T4SS are large multi-subunit complexes involved in substrate transport and pilus biogenesis. The simplest T4SS consists of 11 proteins, named VirB1 to VirB11, after *Agrobaterium tumefaciens* T4SS (Christie et al., 2005, 2014). This macromolecular complex spans across the inner and outer membranes and the periplasm in between. T4SS architecture is well-preserved in most conjugative bacteria, consisting of four distinct sections: the pilus, the core channel complex, the inner membrane platform and the hexameric ATPases that provide the energy for substrate transport and pilus biogenesis (Cabezón et al., 2015). One of them, the traffic ATPase VirB11, was shown to be the target for inhibition by unsaturated fatty acids (Ripoll-Rozada et al., 2016). Here, we will analyze the progress on the different strategies to inhibit the VirB11 ATPase and the rest of the T4SS machinery. The impact of these results on the fight against the spread of antibiotic resistance genes is discussed.

### Strategies for the Identification of Conjugation Inhibitors

Bacterial conjugation has been reported to be inhibited by a variety of compounds. Indeed, chemicals such as heterocyclic compounds, intercalators, acridine dyes, or quinolones were reported to inhibit conjugation (Hahn and Ciak, 1976; Michel-Briand and Laporte, 1985; Molnar et al., 1992; Mazel and Davies, 1999; Nash et al., 2012). However, posterior revisions showed that these molecules were unspecific, mainly affecting bacterial growth or DNA synthesis. Plants are a rich source of bioactive compounds, such as phenolics, which are able to modify bacterial resistances (Oyedemi et al., 2016). Therefore, a current approach consists of isolating molecules from different parts of medicinal plants to discover new inhibitors. By using this approach, two new drugs: rottlerin [5,7-dihydroxy-2,2-dimethyl-6-(2,4,6-trihydroxy-3-methyl-5-acetylbenzyl)-8-cinnamoyl-1,2-chromene] and the red compound (8-cinnamoyl-5,7-dihydroxy-2,2,6-trimethylchromene) were identified as potent antibacterial chemicals against Gram-positive bacteria. These compounds did not hamper Gram-negative bacteria growth but inhibited conjugal transfer of plasmids pKM101, TP114, pUB307, and R6K (Oyedemi et al., 2016). The planar structure of the compounds suggests that the target of these inhibitors might be the DNA replication system but further studies are required to elucidate the mode of inhibition of these agents.

Alternative attempts to inhibit bacterial conjugation have been based on bottom up strategies, targeting essential compounds of the secretion machinery. One study focused on targeting the conjugative relaxase protein, which is the protein that initiates conjugation upon nicking plasmid DNA at the origin of transfer. Due to its key role in plasmid conjugation, relaxases have been considered as potential targets for inhibitors. Some of these potential relaxase-specific inhibitors belong to the bisphosphonates family of compounds, such as etidronate (Didronel) and clodronate (Bonefos) (Lujan et al., 2007). These compounds were reported to be efficient in restraining conjugative DNA transfer. However, these results turned out to be misleading, as these putative inhibitors were found to work as unspecific chelating agents (Nash et al., 2012). An alternative method to inhibit specifically the conjugative relaxase consisted of the expression of specific single chain Fv antibodies (intrabodies) against the relaxase TrwC of conjugative plasmid R388 (Garcillan-Barcia et al., 2007). Expression of these intrabodies in the recipient cell prevented the accretion of the conjugative plasmid. However, the usefulness of intrabodies in practical clinical care is hampered by the need of a transgenic recipient population expressing them. Besides, each intrabody would be specific only against its cognate plasmid.

VirB8 is an essential assembly protein of bacterial T4SS that also acts as molecular target of small-molecule inhibitors (Smith et al., 2012). A high throughput assay based on the restoration of interactions between two split domains of the *Brucella* VirB8 protein allowed the identification of several compounds that inhibited protein-protein interactions (Paschos et al., 2011). One of the most efficient molecules, B8I-2, is a salicylidene acyl-hydrazide derivative, also known to inhibit T3SS (Keyser et al., 2008). Posterior analysis by X-ray crystallography and *in silico* docking of several of these compounds allowed the determination of VirB8 binding site (Smith et al., 2012). Recently, it has been reported that these small molecules also bind TraE, the VirB8 homolog of the conjugative plasmid pKM101, and some of them inhibit plasmid transfer (Casu et al., 2016). Although some of these molecules displayed a low Kd value in *in vitro* binding experiments, no significant impact was observed on plasmid transfer frequencies, with a 10-fold reduction as the strongest effect. Moreover, none of these molecules had an effect on the conjugation of the unrelated plasmid RP4, diminishing the effectiveness of these compounds to overcome antibiotic resistance.

Other alternatives to develop specific inhibitors focused on the conjugative pilus. These appendages are targeted by a variety of bacteriophages that, upon binding to them, enter inside the bacteria cytoplasm. Some of these bacteriophages are specific to conjugative pili and, therefore, have the potential of discriminating between different bacterial species. For instance, filamentous bacteriophages, such as M13, display high affinity for F-type pilus. This interaction is mediated by the phage coat protein g3p. Addition of the soluble N-terminal domain of g3p to F-plasmid containing bacteria resulted in inhibition of conjugation (Lin et al., 2011). Considering that conjugative pili are needed for bacterial cell contact (Anthony et al., 1994), a step required for the spread of antibiotic resistance genes, the use of specific compounds that inhibit pilus formation could result in a powerful strategy to fight against this problem.

In that sense, peptidomimetic small molecules, such as C10 and KSK85, have been found to disrupt T4SS-dependent transport of pathogenic factors, as well as DNA transfer in conjugative *Escherichia coli*. KSK85 acts impeding biogenesis of the pilus appendage, whereas C10 disrupts T4SS activity without affecting pilus assembly (Shaffer et al., 2016). In this case, authors have used a phenotypic screen to identify these two inhibitors. Both compounds have been tested in plasmids pKM101 (IncN) and R1-16 (IncF) but conjugation efficiency is only decreased to 25% and a high concentration of inhibitor is required (150 μM). Therefore, although these compounds are promising scaffolds, new derivatives need to be found to inhibit more effectively the conjugative process.

**FIGURE 1:**
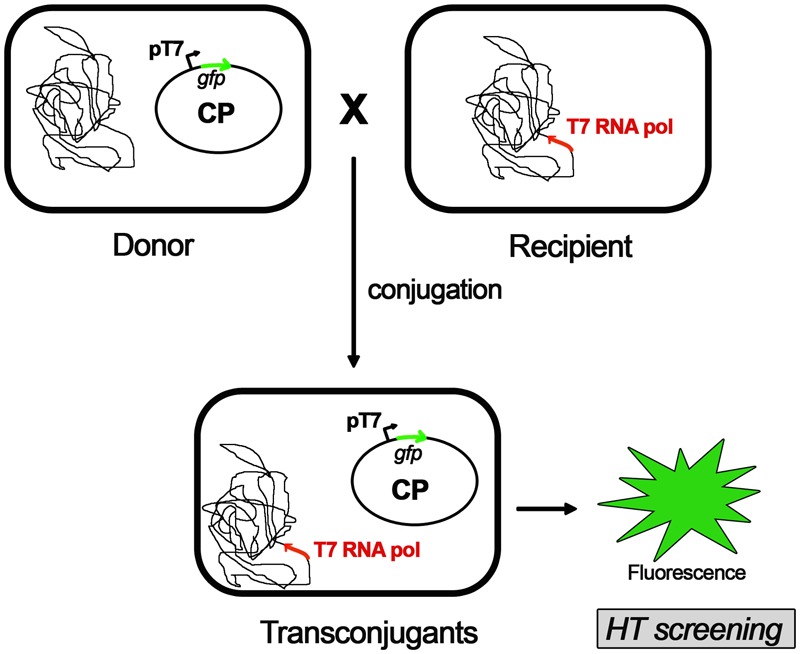
High-throughput conjugation (HTC) assay. A whole-cell automated assay for conjugation based on fluorescence emission of transconjugant cells. A fluorescent protein (GFP) is cloned in the conjugative plasmid (CP) under the control of pT7 promoter and transformed in donor cells. Recipient cells contains a T7 RNA polymerase gene cloned in the bacteria chromosome. Upon mating, the conjugative plasmid is transferred into the recipient cell. The resultant transconjugants will express the GFP after addition of IPTG (isopropyl-β-D-thiogalactopyranoside). Upon induction, the emission fluorescence of GFP is measured, along with the optical densities (ODs) of the cultures. Conjugation frequencies are estimated as a ratio between the fluorescence emitted by transconjugant cells and the total number of cells measured by the OD (for further details on this method, please see Getino et al., 2015).

A novel approach in the pursuit of specific COINs is the screening of potential compounds in whole-cell assays (**Figure 1**). This methodology should be designed in a way that discriminates COINs from false positives affecting cell growth. Thus, the development of luminescence-based high-throughput conjugation (HTC) assays has been shown to be effective in the identification of potential hits. By using HTC assays, unsaturated fatty acids were found to inhibit conjugation of IncF and IncW plasmids without affecting cell growth (Fernández-Lopez et al., 2005). To this end, a library consisting of more than 12,000 natural compounds (NatChem library) was tested. The most effective COIN found in this screening was dehydrocrepnynic acid (DHCA) (Fernández-Lopez et al., 2005). Considering that this compound was extracted from tropical plant seeds (Gussoni et al., 1994) the viability of using it without vast downstream process improvement is limited.

Nonetheless, by using DHCA structure as a chemical template, new synthetic compounds were developed as specific COINs. In particular, synthetic 2-hexadecynoic acid (2-HDA) and other 2-alkynoic fatty acids (2-AFAs) were found to be specific inhibitors of a wide range of conjugative plasmids in different bacteria, including the highly infective and prevalent IncF plasmids (Getino et al., 2015). Furthermore, due to the effect of plasmid burden on host fitness, 2-AFAs could in fact eliminate transmissible plasmids, such as IncF, from bacterial populations. However, other plasmid groups, such as IncN and IncP, were not affected (Fernández-Lopez et al., 2005; Getino et al., 2015).

In addition to 2-AFAs compounds, another family of bioactive compounds was tested in the search for COINs. This collection, named AQUAc, contains more than 1,600 natural compounds. AQUAc was evaluated in order to find potential COINs (Getino et al., 2016). As a result, new COINs were found. Among them, tanzawaic acids A and B were identified as best hits. They specifically inhibited IncW and IncFII conjugative plasmids. The advantage of these compounds is their lower toxicity to animal cells, in comparison to other synthetic COINs. Unsaturated fatty acids (oleic and linoleic acids), 2-HDA, 2-alkynoic fatty acids and tanzawaic acids, all share similar chemical characteristics: a carboxylic group, a long unsaturated carbon chain and the presence of double or triple bonds. These compounds present a 100-fold reduction in plasmid transfer frequencies and, although higher inhibition rates must be achieved to maximize their effectiveness, they constitute key scaffold structures on which to develop more potent and specific COINs. In that respect, knowing the molecular target of these compounds is extremely important, since the use of structure-based drug design (SBDD) methods will allow the design of modified synthetic compounds with higher binding affinities.

### Unsaturated Fatty Acids As Specific Inhibitors of Conjugative Traffic ATPases

Bacterial conjugation is driven by a group of ATPases that empowers almost every step in the conjugative process: DNA unwinding, DNA transport, pilus biogenesis and protein transport (Cabezón et al., 2015). Each of these steps is catalyzed by a specific ATPase, that in the conjugative plasmid R388 are named TrwC, TrwB, TrwK, and TrwD, respectively. TrwC is a protein that nicks the DNA, thus relaxing the conjugative plasmid in an ATP-independent manner (Llosa et al., 1995, 1996). In addition, TrwC displays a DNA helicase activity that results in nucleic acid unwinding (Grandoso et al., 1994; Llosa et al., 1996). TrwB is a DNA-dependent ATPase involved in DNA transfer to the secretion channel (Tato et al., 2005, 2007; Matilla et al., 2010). TrwK is a hexameric ATPase (Arechaga et al., 2008; Peña et al., 2011) that participates in the transport of the pilin molecules from the inner to the outer membranes during pilus biogenesis (Kerr and Christie, 2010). Finally, TrwD is a traffic ATPase that contributes to pilus biogenesis and to DNA translocation (Atmakuri et al., 2004), thus working as a molecular switch between pilus synthesis and substrate transport (Ripoll-Rozada et al., 2013).

Each of these ATPases has been purified to homogeneity and their enzymatic activities have been characterized (Grandoso et al., 1994; Tato et al., 2005; Arechaga et al., 2008; Ripoll-Rozada et al., 2012). The kinetic parameters of each ATPase were analyzed in the presence of the unsaturated fatty acids shown to be efficient COINs (Ripoll-Rozada et al., 2016). Interestingly, only the traffic ATPase TrwD was inhibited by unsaturated fatty acids, such a linoleic acid, and 2-AFAs like 2-HDA. The kinetic parameters for TrwD ATPase inhibition by these fatty acids were determined, indicating that in all cases it was a non-competitive inhibition (Ripoll-Rozada et al., 2016). In contrast, saturated fatty acids, such as palmitic acid, showed no inhibitory effect in conjugation experiments and in ATPase assays.

**FIGURE 2:**
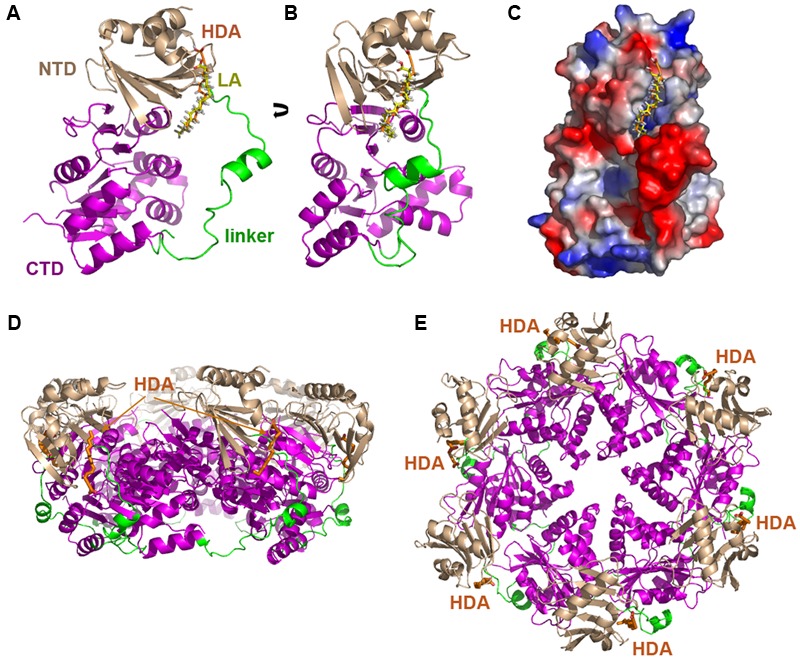
Blind docking of fatty acids in TrwD. Docking predictions between a molecular model of TrwD, the traffic ATPase of the conjugative plasmid R388, and linoleic acid and 2-HDA are shown **(A,B)** (Ripoll-Rozada et al., 2016). These unsaturated fatty acids bind to a pocket formed by the end of the N-terminal domain (NTD, *wheat*) and the linker region (*green*) that connects it to the C-terminal domain (CTD, *purple*). An electrostatic map showing these interactions is included in **(C)**. Inhibitor binding sites in the hexameric TrwD protein are shown in **(D,E)**, corresponding to side and top views, respectively. Docking was performed using the EADock dihedral spacing sampling engine of the Swiss-dock server (Grosdidier et al., 2011).

TrwD belongs to the secretion ATPase superfamily, which also includes members of Type II secretion, Type IV pilus and flagellar biogenesis machineries (Planet et al., 2001). All members of this superfamily are hexameric ATPases, in which each monomer is formed by two domains at the N- and C-termini (NTD and CTD, respectively), connected by a flexible linker of a variable length (Planet et al., 2001; Peña and Arechaga, 2013). ATPase catalysis is driven by swapping this linker over the NTD and CTD (Savvides et al., 2003; Hare et al., 2006). Blind docking predictions (Grosdidier et al., 2011) suggested a putative binding site for uFAs and 2-aFAs located at the end of the NTD and beginning of the linker region that connects it to the CTD where the nucleotide binding site is located (**Figure 2**) (Ripoll-Rozada et al., 2016). These predictions were compatible with a model in which the mode of action of the inhibitors consisted in preventing the swapping movements between the N- and C-terminal domains along the linker region that are required in the catalytic cycle of the protein.

In summary, the discovery of traffic ATPases as potential targets of bacterial COINs opens a promising avenue for the development of new and more potent compounds capable to impair the dissemination of antibiotic resistance genes. However, despite these promising data, the efficacy of these inhibitors to prevent the general spread of antibiotic resistance in naturally occurring environments (Martinez, 2008; von Wintersdorff et al., 2016), hospitals, wastewater systems (Hocquet et al., 2016), agriculture (Capita and Alonso-Calleja, 2013; Economou and Gousia, 2015) and farming settings (Wegener, 2003) needs to be tested. Even today, the main reservoirs where the antibiotic resistance genes arise from and how these genes are rapidly acquired by human pathogens are a matter of debate. COINs could be effective in the discovery of these natural reservoirs. Experiments in controlled microcosms (e.g., freshwater microcosms) and/or experimental animals (e.g., mice gut) should be most instructive. Not only COIN-related experiments will help to identify reservoirs, they will also serve to quantify the dynamics of plasmids in those experiments and the rate at which they can be mobilized to recipient strains that are potential human pathogens. These are some of the exciting goals of ongoing COINs research.

## Outlook

The battle against antibiotic resistance is a challenging problem which is likely to become a progressively increasing burden to our health systems. Several approaches are currently envisaged to fight back against antibiotic resistant bugs. Among them, a promising alternative consists of preventing the propagation of antibiotic resistance genes. Bacterial conjugation is the main mechanism for the wide spread dissemination of these genes. Hence, the search of compounds able to specifically inhibit bacterial conjugation is a preeminent undertaking in the global war against antibiotic resistant bugs. Here, we have reviewed several compounds that are competent in this pursuit. Among these, unsaturated fatty acids and derivatives have been proved to be the most efficient specific COINs. Moreover, the identification of the traffic ATPase TrwD as the molecular target of these uFAs enables the future development of more efficient inhibitors directed against this essential protein of T4SS.

## Author Contributions

All authors listed have made a substantial, direct and intellectual contribution to the work, and approved it for publication.

## Conflict of Interest Statement

The authors declare that the research was conducted in the absence of any commercial or financial relationships that could be construed as a potential conflict of interest.
