# Supplementary material for: Conjugation Inhibitors and Their Potential Use to Prevent Dissemination of Antibiotic Resistance Genes in Bacteria
Source: Front Microbiol. 2017 Nov 30;8:2329. doi: 10.3389/fmicb.2017.02329 (PMC5723004; doi:10.3389/fmicb.2017.02329)
Supplement: Supplementary file 1 [file Data_Sheet_1.pdf]

Supplementary Figure S1

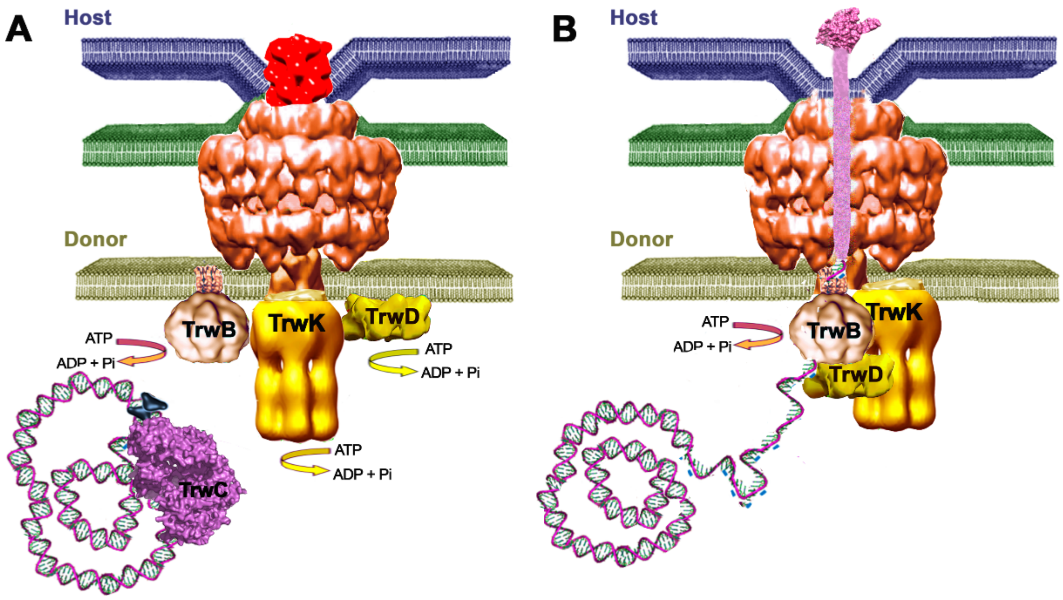

Supplementary Figure S1. Type IV secretion system (T4SS) architecture. Most conjugative systems contain four ATPases involved in the transfer of the conjugative plasmid to the recipient cell (Christie et al., 2014; Cabezón et al., 2015). Two of these ATPases, TrwK and TrwD in the case of the conjugative plasmid R388, are involved in the biogenesis of the T4SS apparatus (*A*). Mobilization of conjugative DNA is mediated by the relaxase protein (TrwC) that nicks the DNA at the origin of transfer and remains covalently bound to the 5´end of DNA (Garcillán, et al., 2009). Another ATPase (TrwB) is involved in coupling DNA processing to the transport of the nucleoprotein complex across the T4SS (*B*). Due to the large size of the nucleoprotein complex (over 900 kDa), it is very likely that the protein crosses the channel in an unfolded state.
